# Interstitial photodynamic therapy as salvage treatment for recurrent head and neck cancer

**DOI:** 10.1038/sj.bjc.6601993

**Published:** 2004-07-06

**Authors:** P-J Lou, H R Jäger, L Jones, T Theodossy, S G Bown, C Hopper

**Affiliations:** 1 National Medical Laser Centre, Department of Surgery, University College London, London, UK; 2 Department of Otolaryngology, National Taiwan University Hospital and College of Medicine, Taipei, Taiwan; 3 Lysholm Department of Neuroradiology, The National Hospital for Neurology and Neurosurgery Queen Square, London, UK; 4 MRI Unit, Department of Imaging, University College London Hospitals, London, UK; 5 Department of Maxillofacial Surgery, University College London Hospitals and the Eastman Dental Institute for Oral Health Care Science, London, UK

**Keywords:** recurrent head and neck cancer, photodynamic therapy, interstitial therapy

## Abstract

Interstitial photodynamic therapy (IPDT) is a technique for applying photodynamic therapy (PDT) to internal tumours using light delivered via fibres inserted percutaneously. This phase I–II study assessed the safety and efficacy of IPDT for patients with persistent or recurrent head and neck cancer unsuitable for further treatment with surgery, radiotherapy or chemotherapy, recruited for ‘last hope’ salvage treatment. Patients were sensitised with 0.15 mg kg^−1^ mTHPC (meso-tetrahydroxyphenyl chlorin) 4 days prior to light delivery from fibres inserted directly into the target tumour (20 J per site at 652 nm) under image guidance. In all, 45 patients were treated. Nine achieved a complete response. Five are alive and free of disease 10–60 months later. Symptomatic relief (mainly for bleeding, pain or tumour debulking) was achieved in a further 24. The median survival (Kaplan–Meier) was 16 months for the 33 responders, but only 2 months for the 12 nonresponders. The only serious complication was a carotid blow out 2 weeks after PDT. No loss of function was detected in nerves encased by treated tumours. Interstitial photodynamic therapy provides worthwhile palliation with few complications and occasional long-term survivors for otherwise untreatable advanced head and neck cancers. It is a treatment option worth adding to those available to integrated head and neck oncology teams.

The treatment of head and neck cancer continues to pose a major clinical challenge. Although some early cancers can be cured, the overall survival rates have remained unchanged for several decades (Black *et al*, 1997). In all, 30–40% of patients with head and neck cancer have persistent or recurrent locoregional disease after completion of definitive treatment. In the majority, surgical salvage is either not feasible or carries a high risk of complications (Watson and Ridge, 1998). Similarly, further radiotherapy is often impossible as surrounding tissues will already have received the maximum tolerable dose of ionising radiation (De Crevoisier *et al*, 1998; Stewart, 1999). Only a very small portion of these patients are long-term survivors.

Photodynamic therapy (PDT) is a site-specific tumour treatment involving the administration of a photosensitiser followed by focal activation in the presence of oxygen using light of a wavelength matched to an absorption peak of the photosensitiser (Dougherty *et al*, 1998). For cancers of the oral cavity, the function and appearance of the mouth can be preserved without sacrificing the efficacy of tumour control (Fan *et al*, 1997; Hopper, 2000). Photodynamic therapy does not have the cumulative toxicity associated with ionising radiation and can be applied safely to previously irradiated tissues. Thus, it has considerable potential for patients with locally persistent or recurrent disease after surgery or radiotherapy.

Photodynamic therapy is usually undertaken with external illumination of the target tissue. Larger lesions in surgically inaccessible sites can be treated with interstitial therapy, using multiple laser fibres inserted directly into tumours through needles positioned under image guidance (Bown *et al*, 2002). In this paper, we report the first phase I–II study to assess the safety and efficacy of interstitial PDT (IPDT) as a salvage treatment for recurrent head and neck cancers.

## MATERIALS AND METHODS

### Patients

Patients with biopsy confirmed persistent or recurrent head and neck cancers were recruited from the multidisciplinary oncology clinic at the University College London Hospitals. These tumours were either considered unresectable or the patients were not suitable for further surgery. Some patients already had complications from prior radiotherapy and none were suitable for further irradiation. All were regarded as having a poor prognosis. All patients underwent either computerised tomography (CT) or magnetic resonance imaging (MRI) to assess the extent of disease. The experimental nature of the treatment was explained and written consent was obtained. The study was approved by the ethics committee of the University College London Hospitals, although during the course of the study, the photosensitising drug used was approved by the European Medicines Evaluation Agency for surface illumination PDT in this group of patients.

### Interstitial photodynamic therapy

Patients were sensitised with 0.15 mg kg^−1^ intravenous meso-tetrahydroxyphenyl chlorin (mTHPC, Foscan®, Biolitec Pharmaceuticals Ltd, Germany) and were treated 4 days later. The light source was a diode laser (Diomed Ltd, Cambridge, or Ceramoptec Gmbh, Germany, wavelength 652 nm) with the primary beam feeding into a beam splitter to produce four treatment fibres (core diameter 0.4 mm, clean cut tip without diffuser). The power delivered down each fibre was 100 mW, checked with a power metre prior to use. Needles (18 G) were positioned transorally or percutaneously into the tumours at approximately 1.5 cm intervals. Ultrasound, CT or MRI guidance was used for needle insertion into deep-seated tumours, particularly for those thought to lie close to vital structures such as the carotid artery, cervical vertebrae or the skull. The fibres were passed through the needles using a flagging system to ensure that the fibre tip was exposed to the tissues and not still inside the needle. Up to four fibres were positioned at a time and a pullback technique was used with 1 cm steps to treat thicker tumours. The energy applied at each treatment site was 20 J. After administration of mTHPC, patients followed a regime of controlled re-exposure to ambient light over a period of 2–3 weeks.

### Assessment and follow-up

The results were assessed 4 weeks after PDT. Complete response (CR): no evidence of disease; partial response (PR): 50% decrease of the tumour volume; stable disease (SD): no change in tumour size enough to describe as PR or PD; progressive disease (PD): an increase of at least 25% in the size of measurable lesions or the appearance of any new lesions. Tumour volume was estimated by physical and radiographic measurements before and after PDT. Most treatments were carried out with palliative intent and the results assessed objectively and subjectively.

## RESULTS

Between July 1997 and December 2002, 45 patients, 42 with recurrent and three with persistent cancers, were treated with salvage IPDT. Details of the patients and their management prior to IPDT are summarised in Table 1

**Table 1:** Patient details^a^

Median age	58 years (range 8–84)
*Sex*	
Male	28 (62 %)
Female	17 (38 %)
	
*Primary treatment*	
Surgery	21 (47 %)
Radiation	41 (91 %)
External only	31
External and brachytherapy	3
Concurrent chemotherapy	7
	
*Salvage treatment after recurrence*	21 (47%)
PDT with external illumination	3
Surgery	13
Brachytherapy	3
Palliative chemotherapy	7

a Many primary and some salvage treatments prior to IPDT included more than one therapeutic modality.

. Tumour details are summarised in Table 2

**Table 2:** Tumour details^a^

**Tumour location**	**No. of patients (no response)**	**Tumour location**	**No. of patients (no response)**
Anterior tongue	8 (1)	Tonsillar region	5
Buccal mucosa	2	Oropharyngeal wall	2
Floor of mouth	4	Maxillary sinus	3 (2)
Alveolar ridge	4 (2)	Nasal cavity	3 (1)
Hard palate	3 (1)	Nasopharynx	1
Tongue base	1	Parotid	2
Neck	6 (5)	Thyroid	1

a In all, 33 were squamous cell carcinomas and three were adenocystic carcinomas. There was one each of: basal cell carcinoma (parotid gland), olfactory neuroblastoma, follicular cell carcinoma, haemangiopericytoma, branchiogenic carcinoma, primitive neuroectodermal tumour, malignant fibrous histiocytoma, osteosarcoma and alveolar soft part sarcoma. Tumour staging at the time of PDT was: rT2:3, rT3:3, rT4:37, rN2:1, rN3:1. The estimated median tumour volume for the entire series was 38 cm^3^ (range 8–224 cm^3^). The figures within parentheses indicate tumours that did not respond to IPDT.

.

In total, 67 treatments were performed on the 45 patients. The laser fibres were inserted transorally, percutaneously or both in 36 (54%), 25 (37%) and 6 (9%) treatments, respectively. Of these, 30 patients (67%) received a single treatment; 10 patients (22%) received two treatments, four patients (9%) received three and one patient received five treatments. Of the treated tumours, 27% (12 of 45) were close to major structures deep in the neck (e.g. carotid artery), another 20% (nine of 45) had invaded up under the base of skull, while 16% (seven of 45) had compressed the trachea. The responses were assessed at 1 month. Complete response was achieved after eight treatments; PR was achieved after 43 treatments, one of which became a CR with additional treatment, as described below, SD was noted after six treatments and PD after 10 treatments (including two who died less than a month after PDT). The overall response rates for patients at 1 month after their last PDT were: CR, nine of 45 (20%); PR, 24 of 45 (54%); SD, six of 45 (13%); PD six of 45 (13%).

**Figure 1:**
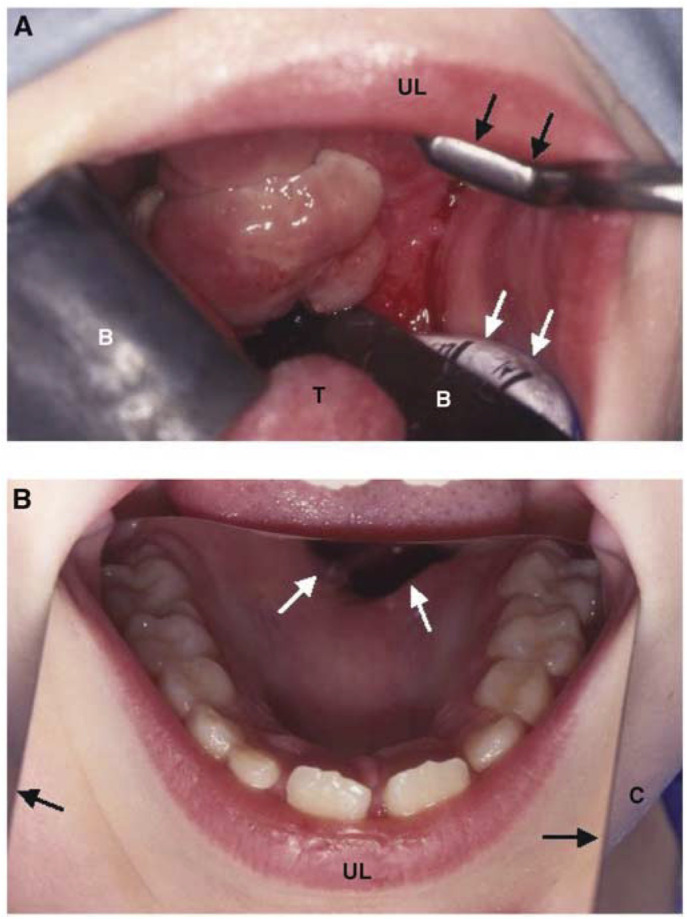
Alveolar soft part sarcoma in an 8-year-old girl. (**A**) Palate viewed immediately before the first IPDT. A retractor (dark arrows) was applied to the upper lip (UL) exposing the bulky tumour that occupied the whole hard palate with extension to the soft palate and the right palatine tonsil. Black shields (B) were used to protect the tongue (T) and normal oral mucosa. White arrows indicate the endotracheal tube. (**B**) Palate viewed 4 weeks after her second IPDT. A mirror was put on her tongue and chin (C) to show the post-treatment condition. Dark arrows indicate the lateral edge of the mirror. There was no visible tumour, although an oronasal fistula (white arrows) had developed. A biopsy at this time showed persistent tumour microscopically, but after a local excision and radiotherapy, she became disease free and has remained well for 5 years.

Although the patients in this study had failed conventional therapy, seven were treated with curative intent because of the relatively early stage of recurrent disease. Four had a CR after IPDT. Two others only had PR and subsequently died of their disease 9 and 17 months later. A young girl with a 5 cm alveolar soft part sarcoma of the hard palate had a limited tumour resection and full courses of ifosfamide, vincristine and actinomycin-D before IPDT, but still had progressive disease. After two treatments with IPDT, only microscopic tumour was detectable. After radiotherapy and a further local resection she became disease free and is now alive and well 5 years later (Figure 1

). Four further patients, who were treated initially with palliative intent, had a CR. Of the nine patients who achieved disease-free status, five are still alive and free of disease 10–60 months after PDT (Table 3

**Table 3:** Summary of complete responders and disease-free patients

**Case no.**	**Age at IPDT (years)**	**Sex**	**Treatment site**	**Pathology**	**Stage original/recurrent**	**Previous treatment**	**Time from initial tx to IPDT (month)**	**Tumour volume (cm^3^) prior to PDT**	**Disease-free time (month)**	**Follow-up time (month)**	**Outcome**
1	52	M	Neck	SCC	N3/N2	R, S	18	12	14	17	LR
2	57	F	HP	SCC	T2/T4	R	30	32	NER	22	UD
3	60	M	TB	SCC	T4/T4	CR, B	46	80	12	17	LR
4	82	F	AR	SCC	T4/T2	S, S	5	8	NER	13	AW
5	21	F	Tongue	SCC	T1/T3	S, S	24	12	26	32	LR
6	52	M	FOM	SCC	T4/T3	S, R	5	10	NER	10	AW
7	59	M	Tongue	SCC	T4/T4	CR	8	60	NER	28	AW
8	42	F	Parotid	ACC	T4/T4	S, R	46	32	NER	15	AW
9^a^	8	F	HP	ASPS	T2B/T2B	C, S	3	20	NER	60	AW

ACC: adenoid cystic carcinoma, AR: alveolar ridge, ASPS: alveolar soft part sarcoma, AW: alive and well, B: brachytherapy, C: chemotherapy, CR: chemoradiation, FOM: floor of mouth, HP: hard palate, LR: died of local recurrence of disease, NER: no evidence of recurrence at the last follow-up, R: radiotherapy, S: surgery, SCC: squamous cell carcinoma, TB: tongue base, tx: treatment, UD died of unrelated disease; IPDT=interstitial photodynamic therapy; PDT=photodynamic therapy.

a Two IPDT treatments were performed to debulk the tumour followed by radiotherapy and local excision for persistent microscopic disease. Since then, she has been free of disease.

).

**Figure 2:**
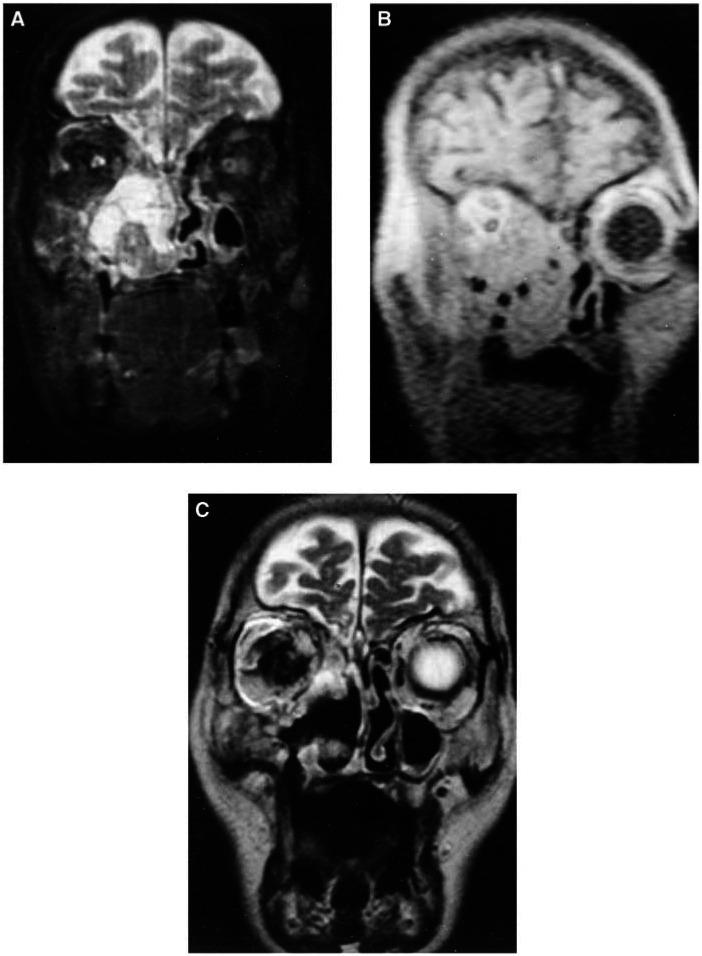
Magnetic resonance images of a maxillary cancer in a 76-year-old man with recurrent episodes of massive nasal bleeding requiring transfusion. (**A**) T2-weighted MRI before IPDT, showing a tumour in the right maxillary antrum and periorbital invasion. (**B**) T1-weighted MRI during treatment showing the position of the MR compatible needles prior to insertion of laser fibers. The needles appear as small, dark (low signal intensity) areas within the tumour mass. (**C**) T2-weighted MRI 6 weeks after treatment demonstrating a dramatic reduction of tumour bulk. Nasal bleeding stopped after IPDT and the patient lived for a further 19 months.

A total of 38 patients were treated with palliative intent, including the four who had a CR. Of the other 34, 21 were fed through a gastrostomy and five also had a tracheostomy. One patient had a tracheostomy without a gastrostomy. In all, 12 had bulky tumours, but were able to maintain oral nutrition. In all but five, the main clinical problem was the tumour bulk (median 48 cm^3^, range 12–224 cm^3^). Some had more than one dominant symptom. It was not possible to improve swallowing or close the tracheostomy in any of these individuals. Nevertheless, PDT reduced the tumour bulk with subjective benefit for the patients in 18 of 29 cases. Bleeding was stopped in three of three, dyspnoea was relived in one of two and brachial plexus compression was relived in one of one, but pain was only helped in two of five cases. Clinical benefit was achieved for 25 of the 40 dominant symptoms. Magnetic resonance images of one patient before and after PDT to reduce bleeding are shown in Figure 2

. Of the 24 patients in this group who achieved worthwhile palliation from PDT, eight survived for more than a year (including two who are still alive, 24 and 31 months after PDT).

**Figure 3:**
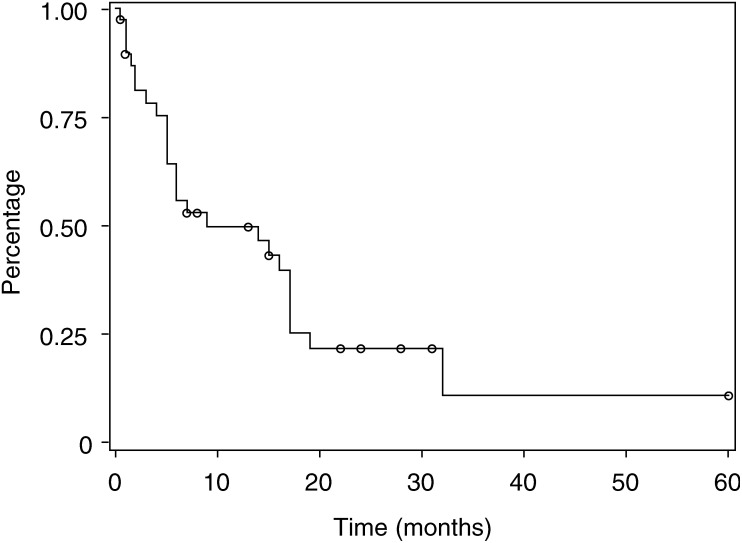
Overall survival (Kaplan–Meier curves).

The 12 patients who did not respond to PDT (stable or progressive disease) were treated mainly to attempt reduction of the tumour bulk (median 56 cm^3^, range 20–224 cm^3^). The location of these tumours is indicated in Table 2. The median survival of this group was only 2 months (range 0.5–6 months). For the whole group of 45 patients, the median survival (Kaplan–Meier) was 14 months (Figure 3

). For the 33 patients responding to PDT, the figure was 16 months. Patients with squamous carcinomas had a slightly shorter survival compared with other pathologies (not significant).

Eight patients had treatments in close proximity to the facial nerve, 15 were close to the hypoglossal nerve and two were close to the brachial plexus. The only neurological deficits prior to PDT were in two hypoglossal nerves and one brachial plexus. After PDT, there was no further loss of function in any of these nerves and in the patient with a brachial plexus deficit, voluntary arm movement improved after PDT.

### Complications

The expected post-treatment pain and swelling subsided in 2–4 weeks. There was no treatment-related airway obstruction. The only major treatment-related complication was a carotid blow out 2 weeks after PDT in a 33-year-old woman with recurrent neck disease. On an MR scan taken 1 month prior to IPDT, the tumour was judged to be close to, but not involving the carotid artery. Post-mortem examination showed malignant cells along the intima of the carotid artery implying tumour invasion. Skin photosensitivity was noted in one patient who failed to comply with the recommended regime for ambient light exposure.

## DISCUSSION

Despite the advances in oncology during the last few decades, most patients with recurrent head and neck cancer still succumb to their disease in a relatively short period of time. The patients in this study were all referred for ‘last hope’ salvage treatment, having been considered unsuitable for further treatment with surgery, radiotherapy or chemotherapy. Nevertheless, five patients (11%) are alive and apparently free of disease 10–60 months later, and the median survival time for the 33 patients (73%) who responded to PDT was 16 months. The short survival of the 12 who did not respond (median 2 months) probably reflects poor patient selection. All these individuals had bulky tumours in areas close to vital structures, which made it difficult to deliver an appropriate light dose to all relevant sites. The most difficult area was the neck. After the carotid blow out (which occurred early in the study), extra care was taken with tumours close to the carotid artery, but this led to a lack of response in five out of six tumours treated in this region. If there is any suspicion that tumours have invaded the carotid artery, PDT is probably not an appropriate treatment.

Damage to cranial nerves and major blood vessels during salvage surgery or radiotherapy is difficult to prevent and greatly reduces the patients’ quality of life. In the present study, the treated tumour encased or was in close proximity to important nerves in 24 patients, but no loss of function was detected and in one case (brachial plexus compression), nerve function improved after PDT. Thus anecdotally, nerves seem to tolerate PDT well. This conclusion is supported by a recent experimental study looking at the effect of PDT with mTHPC on normal arteries and nerves (Kübler *et al*, 2003). This is an aspect that should be studied in further detail, as it is potentially a major attraction of PDT. These unfortunate patients often have serious cosmetic and functional deficiencies due to nerve damage after surgery or radiotherapy, and if this could be avoided, it would be an important step forward in the management of such cases. There is also good experimental evidence that the risk of thrombosis or rupture of normal arteries after PDT is minimal, due to preservation of elastin and collagen fibres (Grant *et al*, 1995). Better imaging should reduce the risk of treating arteries invaded by tumour. The carotid blow-out was our only serious complication. The willingness of patients to undergo repeat treatments showed that the technique was well tolerated.

Our results compare favourably with other therapeutic options. Salvage surgery is rarely curative and carries a 20–40% risk of serious complications (Marcial *et al*, 1980; Rate *et al*, 1991). Similarly, many different salvage radiotherapy regimens have been used, including external beam, interstitial ‘high-dose-rate’ or ‘low-dose-rate’ brachytherapy (De Crevoisier *et al*, 1998; Puthawala *et al*, 2001; Glatzel *et al*, 2002). Although 20–30% of patients may survive for 2 years, moderate to severe complications (including carotid blow-out) have been reported in 30–40% of cases. Chemotherapy for these recurrent cancers has a response rate of no more than 10–30% with a median survival of about 6 months (Clavel *et al*, 1994). Newer drugs have not improved the response rates significantly from those seen with traditional cisplatin-based chemotherapy (Forastiere *et al*, 1998).

Two of the three sarcomas treated in this study responded remarkably well. The young girl with a sarcoma of the palate is free of disease at 5 years and the older patient with a skull base osteosarcoma had good relief from his headache. The third patient, a boy of 15, did not respond and died a few weeks after treatment. PDT may be able to play an important role in the management of these rare tumours, especially those occurring in surgically inaccessible sites, as the response to chemotherapy and radiotherapy is not always good (Casanova *et al*, 2000).

Recurrent head and neck cancers are difficult to treat because of the complex regional anatomy and close proximity to vital structures. This study shows that IPDT can be an effective salvage measure for these unfortunate patients. It is minimally invasive, effective for carcinomas and sarcomas and can be used in patients who are unfit for further radiotherapy or surgery. It is a therapeutic option that is likely to become a useful addition to the armamentarium of the integrated head and neck oncology team for late-stage disease.
